# The Statistical Value of Raw Fluorescence Signal in Luminex xMAP Based Multiplex Immunoassays

**DOI:** 10.1038/srep26996

**Published:** 2016-05-31

**Authors:** Edmond J. Breen, Woei Tan, Alamgir Khan

**Affiliations:** 1 Australian Proteome Analysis Facility (APAF), Level 4, Building F7B, Research Park Drive, Macquarie University, Sydney NSW 2109 Australia; 2 Bio-Rad Laboratories Inc. 2000 Alfred Nobel Drive, Hercules, CA 94547 USA

## Abstract

Tissue samples (plasma, saliva, serum or urine) from 169 patients classified as either normal or having one of seven possible diseases are analysed across three 96-well plates for the presences of 37 analytes using cytokine inflammation multiplexed immunoassay panels. Censoring for concentration data caused problems for analysis of the low abundant analytes. Using fluorescence analysis over concentration based analysis allowed analysis of these low abundant analytes. Mixed-effects analysis on the resulting fluorescence and concentration responses reveals a combination of censoring and mapping the fluorescence responses to concentration values, through a 5PL curve, changed observed analyte concentrations. Simulation verifies this, by showing a dependence on the mean florescence response and its distribution on the observed analyte concentration levels. Differences from normality, in the fluorescence responses, can lead to differences in concentration estimates and unreliable probabilities for treatment effects. It is seen that when fluorescence responses are normally distributed, probabilities of treatment effects for fluorescence based *t-tests* has greater statistical power than the same probabilities from concentration based *t-tests*. We add evidence that the fluorescence response, unlike concentration values, doesn’t require censoring and we show with respect to differential analysis on the fluorescence responses that background correction is not required.

Multiplexed immunoassays have widespread applications in the life sciences^1^ and as employed here are often used for detecting key biomarkers such as those associated with human inflammation responses. Yet for life scientists, immunoassay data analysis on low abundant analytes gets confounded because concentration detection limits censor many of their readings from analysis. In this report we show censoring is a concern for concentration based analysis and not for fluorescence based analysis and it is seen that fluorescence based analysis has higher statistical power than concentration based analysis and therefore a better choice for assigning statistical significance to main effects.

Censoring prevents fluorescence responses outside the range of specific standards from being assigned a concentration value. Universally, concentration based analysis are reported in the literature^2^,^3^,^4^,^5^,^6^,^7^,^8^,^9^,^10^,^11^,^12^ and analysis treating out-of-range values simply as unreliable are increasing the risk of obtaining inaccurate concentration estimations and false conclusions^13^,^14^,^15^,^16^. Therefore, in concentration based analysis out-of-range values at times are imputed by maximum likelihood estimations (MLE)^5^,^17^,^18^,^19^, extrapolation^3^, or substitution^20^. Extrapolated values are generally those estimated concentration values that are out-of-range of the standards but are still within the limits (top/bottom) of a fitted five or four point logistic curve^21^. MLE fits a distribution for both the values for detected observations and the proportion of out-of-range values and is considered reliable if the number of in-range values is large, but can be sensitive to outliers^22^. However, what is not clear to many is that fluorescence based analyses are free of such concerns simply because they don’t have out-of-range problems.

There is a ubiquitous appeal for expressing immunoassay results in terms of concentrations that comes from the premise they provide absolute quantification, allows the reporting of sensitivity in well understood terminology; i.e., pg/ml or ng/ml, and is needed for dosage determination. While the underlying fluorescence responses are assumed to provide only relative quantification of the analyte abundances. This assumption may not be strictly true for the observed fluorescence responses for a given platform; as automatic calibration steps during setup yields a highly reproducible signal output even across different instruments^23^. Recently, we have argued that for statistical differential analysis and for reproducibility fluorescence values are the better choice and they alleviate the concern of determining levels of detection^15^,^17^,^22^,^24^,^25^. It this report a xMAP suspension bead-based immunoassay format is used to demonstrate fluorescence based data analysis, however the methodological techniques used here are generic and applicable to other immunoassay platforms that use typical sigmoidal/ logistic concentration curves to map fluorescence to concentration values.

## Results

### Blanks and standards

**Figure 1:**
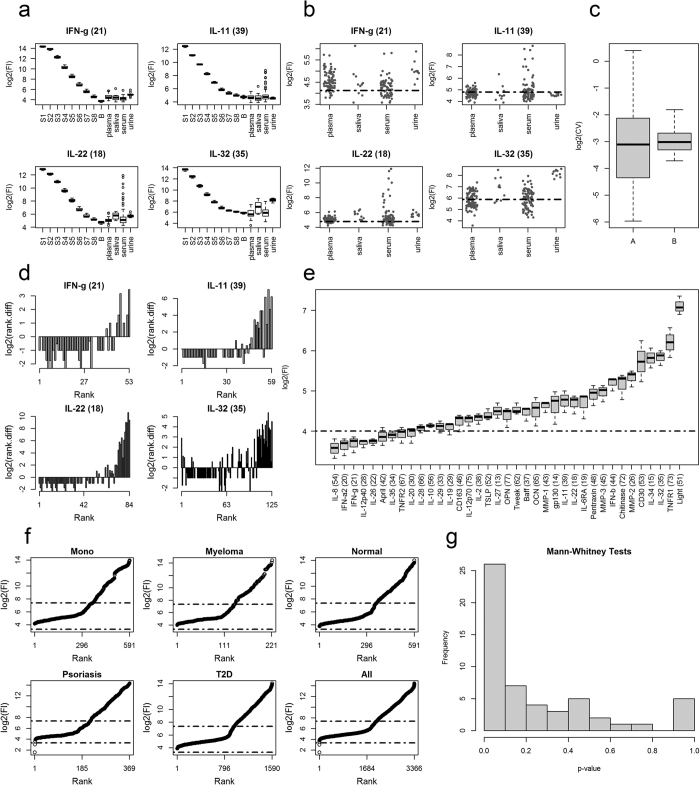
Fluorescence LOD analysis. (**a**) Tissue test sample analyte fluorescence distributions against the associated standards (S1, …, S8) and blanks (B). Standards represent the fluorescence responses obtained from a set of standards of known concentrations for each analyte under investigation. The blank represents the test kits fluorescence response when its target analyte is missing. The background matrix used for the standards and blanks is typically, and as here is, the assay’s diluent. (**b**) Tissue fluorescence scatterplots for 4 analytes. The dashed horizontal line in each plot in (**b**) is the threshold (censor) determined by Bio-Rad’s Manager Software as the lower limit of detection. (**c**) Box plot Log2 distributions for the fluorescence response coefficient of variation (CV) above (A) and below (B) the associated LOD. The log of the values is used because there are two large outliers in the above (A) distribution. (**d**) The log2 of the rank differences as a function of rank; that is: ***rank***.***diff***(***r***) = ***Fl***(***r ***+ 1) − ***Fl***(***r***); where *r* is a *rank*, an integer value, from the set: ***r***∈{1,…, ***n−1***}, and where ***n*** is the number of ranks in the set **1:n**. Rank assigns to each unique fluorescence response an ordering from lowest to highest response such that ***Fl***(***rank***) < ***Fl***(***rank *****+** **1**) and where ***Fl***(***rank***) is the fluorescence response associated by rank. (**e**) Analyte blank response distributions in rank order. The dashed horizontal line represents the level at which most if not all the patient fluorescence responses are above. (**f**) Patient sample fluorescence responses for plasma in rank order and according to condition. The lower and upper dashed horizontal lines represents the median response from the minimum (IL-8) and maximum (Light) blank responses. Abbreviations: **Mono** = mononucleosis, and **T2D** = type 2 diabetes. (**g**) Histogram of 54 Mann-Whitney test p-values obtained from cytokine tissue pairwise comparisons for the 9 cytokines that have median test sample response less than its lowest standard (S8): IFN-g (21), IL-10 (56), IL-11 (39), IL-12p40 (28), IL-2 (38), IL-20 (30), IL-22 (18), IL-28 (66), IL-32 (35).

Comparing patient fluorescence responses against standards and blanks reveals whether the patient responses are within range of the standards^14^ (Fig. 1a). The same distributions for all 37 analytes are given in Supplementary Figure S1. The analytes in (Fig. 1a) were chosen because their sample median fluorescent response was below the median response of their lowest standard (S8). For IL-11 its tissue median response is actually below the median of the blank (B, Fig. 1a). Also note, the median response for IL-32 in plasma and serum are also below their median blank response.

It’s not uncommon for test sample fluorescence responses to be less than their blank^2^,^14^. Given that analyte distributions with a median response below the blank appear normal against the shape of the distributions seen for other analytes, it’s arguable what the real association is between the standards and test samples^14^. While fluorescence responses below the associated blank cannot be assigned concentration values without using imputation^19^, they can be used for differential analysis and for assigning statistical significances to treatment effects.

### Fluorescence and concentration level of detection

When working with concentration values a decision is made on how to set the appropriate level-of-detection (LOD) for each analyte^25^. However, the inverse procedure of going from fluorescence to concentration, via the inverse 5PL curve, also has limits (top/bottom), beyond which no concentration values can be obtained. In contrast, when working with the fluorescence responses no level of detection is generally specified^2^,^13^,^26^,^27^,^28^,^29^,^30^,^31^,^32^,^33^. This is because fluorescence enables the analysis of low signal and will have more power for testing differences in analyte expression^13^,^14^. In this report we are interested to test if censoring the fluorescence responses is required.

If a fluorescent level of detection exists it is expected that as the fluorescence response drops, the responses, for a given analyte, should approach some lower limit. This was not seen in the analyte responses that have a median response less than their lowest standard (Fig. 1b). When the individual fluorescence responses are compared against their lower level of detection, LOD, (Fig. 1b: dashed horizontal lines) as obtained from the Bio-Plex Manager^TM^, there doesn’t appear to be a real difference in point scatter above or below these thresholds (Fig. 1b). While IL-22 appears to show a greater scatter above its LOD, this is only a perceptual limit and comes from the scaling to view all the responses for the serum samples with respect to the other analytes. Note, for IL-11 all the urine samples are below the associated LOD (Fig. 1b). Table 1 gives the coefficients of variation (CV), used here as a standardised measure of point scatter and dispersion, for analyte responses with respect to tissue types that have at least 5 fluorescence responses above and below their associated LOD. The samples for analysis were chosen such that the N nearest points above and below the LOD was selected. Therefore, in total 2N points for each analyte and tissue combination was used. The actual value of each N was determined from the minimum number of responses either above or below the associated LOD (Table 1). If the analyte responses approach a lower limit then we expected on average that the point scatter (CV) below the LOD to be less than above it. A test of equality of two coefficients of variation is reportedly equivalent to testing for equality of variances between the logarithmic transformed data^34^. Therefore, we used an *F*-test and the NULL hypothesis that the ratio between the variances of the log2 transformed data above and below the LOD should be one. Seven of the 17 comparisons were found significant at the 0.05 level (Table 1). Of these only two represented the case where the CV was greater above the LOD than below. The boxplot distributions of the log2 of the CVs (Fig. 1c), for the groups above (A) and below (B) the LOD (Table 1) shows that above the LOD the CVs have a greater range of values than below it. However, statistically the two groups (A, B), according to a Mann-Whitney test they are statistically similar (w = 135, *p-value* = 0.65) and similarly according to a paired *t-test* (t = −0.42, df = 16, *p-value* = 0.68). Thus, confirming our original observation that there appears little difference in point scatters above and below the LOD, as determined from the standards.

A lower limit wasn’t revealed either by considering the log2 of the rank differences; where if a limit of detection existed then this difference should approach zero as the rank reduces (Fig. 1d). Over the first 50% of the differences the average log2 difference for all these analytes is approx. −1 (Fig. 1d). This reflects ½ a fluorescence response unit change per unique fluorescence response change; and suggests that *Fl*(*rank *+ 1)−*Fl*(*rank*) = 0.5, when *rank* is in the set 1:(*n/2*) and *n* is the largest rank value (Fig. 1d).

Looking at the ranked ordered of the analyte blank distributions (Fig. 1e) shows a fairly continuous range of values. The ranked fluorescence responses for all the patient plasma samples (Fig. 1f) reveals a log2 value of 4 may indicate a potential lower level of detection. However, this is just the response level that most patients’ responses are actually above. This ordering also exposes the existence of a bend, or dog leg, near the fluorescence log2 value of 6 (Fig. 1e). While this bend is not discussed here, it is noted that it represent approximately the median fluorescence response, as about 50% of the fluorescence responses fall below the log2 value of 6. As there are 8 of the 37 analytes with a median blank response below the log2 value of 4 (Fig. 1d; dashed horizontal line), reveals that the Luminex100 instrument has the ability to distinguish fluorescence signal weaker than that observed for the majority of our test samples.

While, the above observations suggest that there is no detectable lower level-of-detection for the fluorescence responses given here, it doesn’t rule out the case that the signal observed for the low abundant analyte samples represents just noise. If this was the case then the *p-value* distribution obtained for the low-abundant analyte pairwise tissue comparisons (Fig. 1g), should appear uniform^35^. However, as seen (Fig. 1g) there is an overrepresentation of the lowest *p-values*, indicating the presence of structure and grouping effects in the low abundant responses with respect to fixed analyte and pairwise tissue comparisons.

### Differential analysis

For differential analysis of analytes across treatment, tissue or disease statuses, a variety of statistical approaches can be used, such as: *t-tests*^36^,^37^, ANOVA and ANCOVA^37^,^38^, and nonparametric tests^12^,^24^. However, mixed-effects modelling with fixed and random-effects are emerging as the preferred approach^5^,^14^,^27^,^39^. This is because of its ability to represent different experimental designs, to cope with unbalanced data sets, handle paired and longitudinal information and each random effect decreases the degrees of freedom less than the same effects when treated as fixed^40^, leaving more degrees of freedom for the additional effects and error term estimations^41^.

The most common method for employing these tests is to consider one analyte at a time using multiple statistical models^42^. While this has the advantage of computational tractability, compared to a single global statistical model approach, it has the disadvantages of not incorporating the variances from all the analytes simultaneously, it will missing intrinsic expression similarities or differences and theoretically increasing false positive and negative rates^27^,^42^. Other advantages for including all the analytes in a single statistical model are that it allows for easy discovery of statistically significant interactions^43^, and provides a simple framework for extracting corrected and uncorrected probabilities for analyte differences across, or in pair-wise contrasts, between tissues/treatments and disease status.

A common form of data reduction prior to statistical analysis is the stratification of results such that only the patients with conditions or tissues/treatments of interest are included in the statistical models. For example: to compare the Mononucleosis patients against the normal patients, Supplementary Table S1, two statistical models would normally be used, one including just the plasma data and other including just the serum data. However, doing so is overlooking the ability to correct for tissue and disease differences as needed; thereby, allowing more data into the analysis and increasing its underlying statistical power.

**Figure 2:**
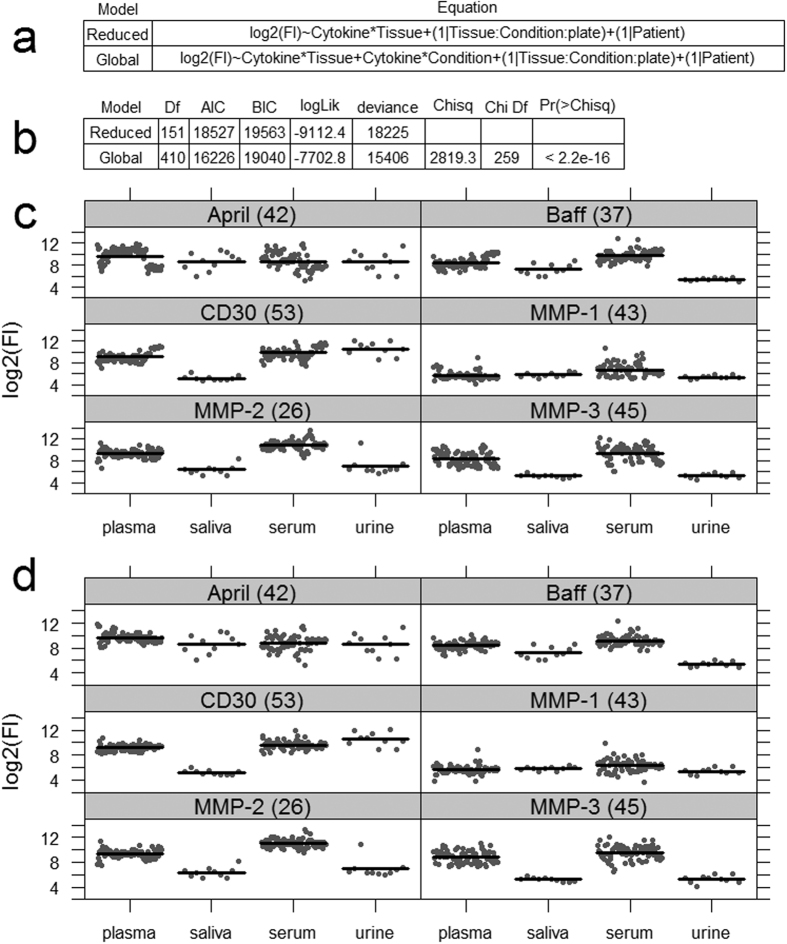
Statistical model for analyte expression. (**a**) Two linear mixed-effects models in R-notation. Main fixed effects are Cytokine, 37 levels, Tissue, 4 levels, and Condition with 8 levels. (**b**) Gives the results of a statistical comparison of the reduced model against the global model. AIC Akaike’s Information Criterion, BIC Bayesian Information Criterion, and the smaller they are the better the model fit. Comparisons between reduced, (**c**), and global mixed-effects models (**d**) for selected analytes using regression conditional plots. Conditional plots show the relationship between the outcome and explanatory/conditional variables to be viewed as other effects are held constant. Note for the global model, (**d**), the cluster of points (residuals) around each conditional mean response (dark horizontal line) is tighter than that seen in the reduced model, (**c**). Both models contain the same number of samples per tissue. For brevity, only results for 9 of the 37 analytes are given, however, the same comparison but for all 37 analytes are given in Supplementary Fig. S2. Since the conditional residuals are reasonably scattered above and below their respective means, implies that either model is a reasonable fit to the data.

Here, two linear mixed-effects models are first examined (Fig. 2a), both use all the available data, but in one (the reduced model) not all the data associations are included. In both models the log2 of the fluorescence responses (Fl) are modelled with two fixed main effects (reduced model) or three fixed main effects (global model) plus 1^st^ order interactions, Tissue:Cytokine and/or Condition:Cytokine. Both models include the same two scalar random-effects defined by the terms in brackets and which are conditional, ‘|’, on Plate:Condition:Tissue or on Patient. The first random-effect allows for tissues and conditions to associate across different plates, and from the data sets here there are 14 such associations, see Supplementary Table S1. The 2^nd^ random effect accounts for patient-to-patient variations. Both these models adjust for extraneous variations due to plates, conditions, tissues and patient differences and because we have sparse data, Supplementary Table S1, we avoid overfitting by not including higher order fixed-effects interactions such as, Cytokine:Tissue:Condition.

For fluorescence, the advantage of including more data associations in the statistical analysis can be tested easily; by testing if the global model adds explanatory value over the reduced model and where the NULL hypothesis is no explanatory value added. From such analysis (Fig. 2b) we see while the global model uses nearly 3 times the degrees of freedom (Df) there is good statistical reasons (*p-value* < 2.2e-16) to use a global model over a reduced model as in this case it helps explain more variation in the data. This can also be visualized using regression conditional plots^44^ and in terms of a reduced spread in the partial residuals about the conditional means for each analyte obtained from the global model compared to the same results obtained from the reduced model (Fig. 2c,d). Therefore, all statistical analysis here will use the global model (Fig. 2a).

### Statistical significance of analyte differences

For the fluorescence responses there are no issues when applying the above statistical models (Fig. 2a) because of a reasonable number of patient readings for each analyte against tissue and conditions (Supplementary Fig. S3). In contrast, for the concentration analysis there were major problems due to zero readings per analyte against tissue or condition (Supplementary Fig. S3). For the concentration data set there are 4 analytes IL-11, IL-34, Light, and MMP-3 that have at least one cell with zero patient readings (Supplementary Fig. S3) and the mixed-effects models in (Fig. 2a) need at least 1 entry per cell to avoid matrix-rank deficiency problems during analysis. Therefore, these 4 analytes were removed from consideration for the concentration analysis, but not from the fluorescence based analysis.

**Figure 3:**
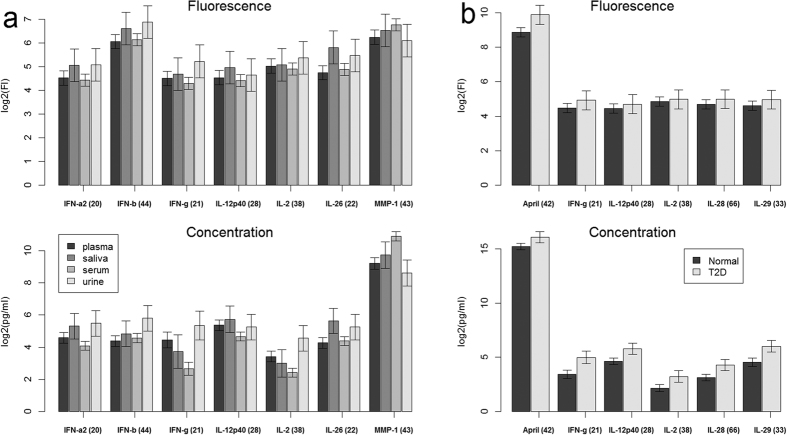
Comparison of analyte expression fluorescence and concentration levels. Adjusted means obtained for selected analytes from the global mixed-effects models. (**a**) Analyte means adjusted for condition, plate and patient differences across tissue. (**b**) Analyte means adjusted for tissue, plate and patient differences across conditions: Normal and T2D (Type 2 Diabetes). Error bars represent 95% confidence.

The probabilities of analyte abundance changing across tissue types are easily obtained from a mixed-effects model and are given in Table 2. For completeness, the probabilities for the 6 pairwise comparisons between tissue types per analytes, for all analytes, are given in Supplementary Table S2. The Sig. column, Table 2, highlights the analytes that have significant abundance changes across tissue types according to either fluorescence responses or concentration values. The concentration analysis shows that nearly every analyte except for IL-10, IL-12p70 and TSLP, are significant at the 0.05 level. Seven analytes (IFN-a2, IFN-b, INF-g, IL-12p40, IL-2, IL-26 and MMP-1) are significant according to concentration but not according to fluorescence, Table 2. By looking at the adjusted means and the 95% confidence limits against tissue (Fig. 3a) for these analytes it’s seen for fluorescence that all confidence limits for each of the 7 analytes, across tissues, overlap each other. This is not so for the concentration responses and this is why theses analytes show up as being significant according to concentration but not according to the fluorescence analysis.

In Table 3, analyte pair-wise comparisons between normal patients and diseased patients (COPD, Mono, Myeloma, Psoriasis, RA, Sepsis and T23) are given. For brevity, Table 3 shows only those comparisons that are significant according to either the fluorescence or concentration based analysis at the 0.05 level. There are 56 comparisons in Table 3, 39 are significant according to fluorescence, but 45 according to the concentration results. The largest difference between the fluorescence and concentration results is seen for the pair-wise differences between normal and T2D patients. For this comparison: the fluorescence result shows only April as being differentially expressed, while from the concentration analysis IFN-g, IL-12p40, IL-2, IL-28 and IL-29, are identified as being differentially expressed. From the adjusted means and the 95% confidence limits according to the fluorescence and concentration responses for these analytes (Fig. 3b), it is clear from the concentration data that these analytes, except for April, are differentially expressed between normal and T2D patients, but equally clear from the fluorescence analysis is, that these same analytes are not differentially expressed (Fig. 3b).

We believe that the differences between the relative analyte abundances seen between the concentrations and fluorescence responses (Fig. 3) can only come about if the mapping from fluorescence to concentration changed the relative abundances and differences between the analytes from that given by the fluorescence responses.

### Mapping the fluorescence response into concentration values

**Figure 4:**
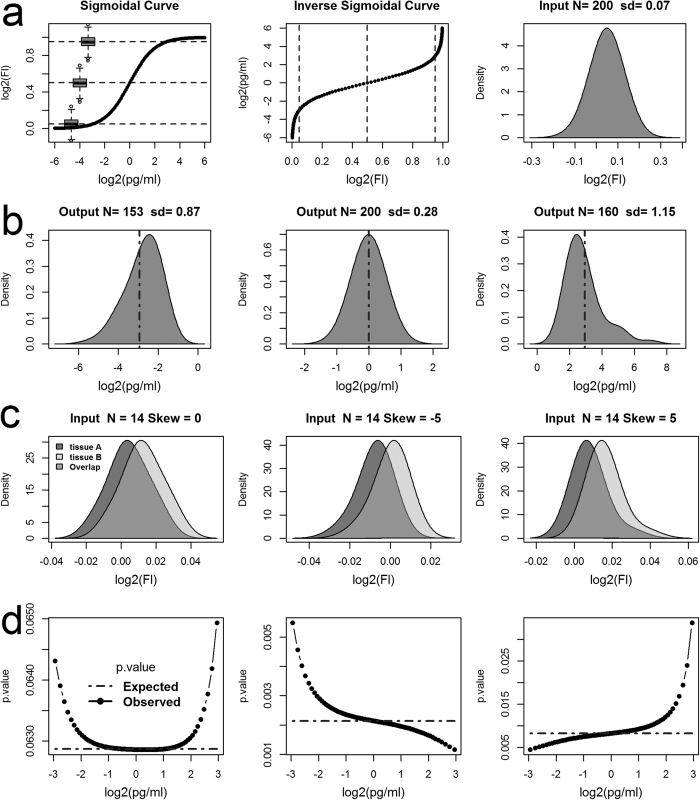
Modelling the mapping of fluorescence responses to concentration values. (**a**) A Simulations of mapping a hypothetical fluorescence response (Fl) distribution to concentration values (pg/ml) through a sigmoidal curve. (**a**) Gives the concentration response curve (sigmoidal curve), the corresponding inverse sigmoidal curve and the input fluorescence distribution. Note, the log2(Fl) responses are normalized to lie between 0 and 1. The boxplots overlaid on the sigmoidal curve show the relative fluorescence range/scale of the input distribution at low (0.05), middle (0.5; EC50 response) and high (0.95) responses levels against the concentration curve (for comparison see Supplementary Fig. S1). The dashed horizontal and vertical lines on the sigmoidal and the inverse curve respectively highlight these levels. (**b**) Shows the corresponding output concentration distributions after mapping the input distribution in (**a**) to pg/ml when the input distribution is centred at low, middle, or high log2(Fl) responses. The vertical dashed lines overlaid on the output distributions represent the expected mean concentration as obtained from the input mean fluorescence response. (**c**) Simulations of mapping the log2(Fl) responses from two hypothetical tissues A and B to pg/ml is given. Three input distribution pairs that differ only with respect to their level of skewness (0, −5, and 5 respectively). The distance between the input means in each pair was set to achieve a Cohen’s effect size of 0.8. (**d**) Gives the resulting *p-values* obtained from two-sample *t-tests* on the output concentration distributions, as the input distributions (**c**) are mapped to concentration values from low (0.05) to high (0.95) response levels; step size approx. 0.01 log2(Fl) units. Note, the expected *p-values* in (**d**) represent the results obtained from *t-tests* on the input fluorescence distributions after each translation along fluorescence axis.

It should be noted that the differences between the fluorescence and concentration results shown above are mostly highlighting low abundant analytes (Fig. 3). Yet it is the low abundant analytes that at times are the most interesting to the life scientists and can occupy most of the analysis. Therefore, simulation is used to confirm our understanding of the differences between the analyte abundance profiles seen in (Fig. 3). Mapping a hypothetical fluorescence response distribution to concentration values through a simple sigmoidal curve (Fig. 4a) at low (0.05), middle (0.5; the EC50 response) and high (0.95) response levels, reveals that the resulting concentration distributions are skewed either to left for low fluorescence response, or skewed right at high fluorescence response level (Fig. 4b). EC50 represents the effective concentration that produces 50% of the maximum fluorescence response. Note also, not all input responses were mapped to concentration values because the maximum (top) and minimum (bottom) log2 fluorescence responses that can be mapped via the inverse sigmoidal curve in this simulation is 1 and zero respectively. Also note, the variances, as measured by the standard deviation, *sd*, increases as the output distribution moves away from the EC50 location. This implies that any resulting statistical test on the concentration distributions when the fluorescence distribution is normally distributed but not centred at the EC50 response will have less statistical power and hence higher *p-values* compared to the input fluorescence responses.

Simulations for mapping the fluorescence responses from two hypothetical tissues/treatment groups A and B to concentrations are used to see how censoring and mapping fluorescence responses to concentration values effects statistical comparisons (Fig. 4c,d). Three input distribution pairs are used and they differ only with respect to level of skewness (Fig. 4c). The resulting *p.values* from two-sample *t-tests*, performed on the input fluorescence responses after translation, along the fluorescence axis, and on the corresponding output concentration distributions after the translated fluorescence responses are mapped to concentration values. This was carried out in a stepwise fashion (stepsize = 0.01) across the range of responses from 0.05 to 0.95 inclusively (Fig. 4d). The left plots (Fig. 4c,d) gives the case when the input distributions are normally distributed (skew = 0), and shows that the resulting concentration *p.values*, increase with distance from EC50 location (0 on the log2 scale). However, the expected *p.values* as obtained from the translated fluorescence responses remains constant. The middle plots (Fig. 4c,d) gives the case when both input distributions are skewed left (skew = −5) and reveals that below the EC50 concentration the concentration *p.values* increase, but above the *p.values* decrease. The right plots (Fig. 4c,d) show the opposite case, when the input distributions are skewed right (skew = 5), and below the EC50 the concentration *p.values* are seen to decrease but above they increase.

The plots in (Fig. 4d) reveal that the concentration *t-tests* results changed with respect to concentration, implying that the effect-size between the inputs must also be changing with respect to concentration. Therefore, the underlying relative abundances determined from concentration based analysis can be different from that obtained from a fluorescence based analysis. Also note, that the expected *p.values* (Fig. 4d), obtained from the input fluorescence responses remained constant with respect to translation along the fluorescence axis, telling us that the results of *t-tests* are invariant under translation and more importantly, that differential analysis performed on the raw fluorescence values don’t actually require any form of background correction prior to analysis.

## Discussion

Here we have added evidence that there is no need to specify a limit of detection for the fluorescence response. That a global statistical model has more statistical power than a reduced statistical model, and this we believe extends to the cases of looking at single analytes at a time or via stratifying the results into logical groupings and analysing these groups individually. It was demonstrated that the observed concentration values and resulting *p.values* from comparative concentrations based analysis are affected by the shape of the input fluorescence response distributions and the actual location on the concentration curve these input distributions map through. Because of these relationships the exact output probabilities after mapping fluorescence responses to concentration values is to some extent unpredictable, even if the input fluorescence comparative probability is known. The output probabilities according to concentration comparisons can be less than obtained from the same comparisons using the fluorescence responses. This then leads to false conclusions and claims of greater effect size and significance. In other instances the output concentration comparisons probabilities can actually be greater than that obtained from the input fluorescence distributions leading to the false conclusion of little or small observable effect size.

Here it was seen that the analyte blanks showed varying response levels, suggesting that non-specific binding of multiplexed detector antibody complexes might be contributing to this variations. Some bead types appear to be inherently stickier, such as Light (51), while other bead types appeared much less sticky, for example IL-8 (54). We suggest that this non-specific binding observed in the blanks maybe blocked by agents found in the test sample^14^. These blocking agents could be lipids, cholesterol, proteins, or heterophilic antibodies. However, the common approach of subtracting the blank from the test sample responses to correct for nuisance background levels must assume no blocking is occurring in the test samples, yet it’s not unusual to find sample responses for low abundant analytes less than the associated blank and this would not be the case if this assumption was true. Alternatively, by not subtracting the blank, as done here, assumes 100% of the non-specific binding sites are blocked by agents in the test samples. The reality, perhaps, will be between these two extremes. Of concern, for the analyst, is that individual estimates of the background levels will be mixed in with noise. Subtracting these estimates especially from the low abundant analytes will result in values with even more noise. Alternatively, the effect of no background subtraction will mean slightly higher response values for the low abundant analytes and will underestimate slightly any fold change measurements. Yet for differential analysis this isn’t a problem because it was shown here via simulation that background levels should have no impact on differential analysis when using the fluorescence responses. This is because our simulations revealed that *t-tests* were invariant under translation; that is, *t-test*(*x,y*) = *t-test*(*x* + *b*, *y* + *b*); where *x*, *y* represent two groups of values and *b* represents the background level.

The use of fluorescence response in the case of xMAP technology permits data analysis to be performed independently from a standard curve. As a general guideline, for data points collected in duplicate or triplicate wells, intra fluorescence responses %CV reflects actual well-to-well variation in reading. This %CV value is typically smaller than intra-observed concentration %CV^14^, which is dictated by the precision of the entire standard curve. As a result, a poor between-plate %CV on a standard curve will have a larger impact on data interpretation if sample analysis is concentration driven. This is not an issue if data analysis is performed using fluorescence response. In addition, sample analysis in the absence of a standard curve will also help to maximize the number of samples to analyse per 96-well plate, from 39 to 47 samples (run in duplicate wells).

## Methods

From 169 patients, a total of 191 samples, for either one or two of four possible sample types (plasma, saliva, serum and urine) were purchased from Bioreclamation Inc (Hicksville, NY). All blood specimens were collected at clinical locations using standard vacutainer-type blood collection tubes and processed to plasma or serum by the vendor. The samples were aliquoted and stored frozen at −80 °C for single use. Bioreclamation Inc. collects every sample under IRB approved protocols, where ethical guidelines are followed to protect patient confidentiality and safety. Each sample has the patients consent for use in a wide range of research including the development of commercial products or services.

Patients were classified as either normal or as having one of seven possible disease states (COPD, Mononucleosis, Myeloma, Psoriasis, Rheumatoid Arthritis, and Type 2 Diabetes). Patient personal record and medical history were blinded and their sample identification was randomized prior to analysis. Samples were analysed across three 96-well plates, labelled here as Plate = (plate1, plate2, plate3), for the presences of 37 analytes (April, Baff, CD163, CD30, Chitinase, gp130, IFN-a2, IFN-b, IFN-g, IL-10, IL-11, IL-12p40, IL-12p70, IL-19, IL-2, IL-20, IL-22, IL-26, IL-27, IL-28, IL-29, IL-32, IL-34, IL-35, IL-6RA, IL-8, Light, MMP-1, MMP-2, MMP-3, OCN, OPN, Pentraxin, TNFR1, TNFR2, TSLP, and Tweak). The actual number of patients associations between sample types, conditions and plates are given in Supplementary Table S1. Note, the 16 mononucleosis patients across plasma and serum are paired, as is the 6 myeloma patients across plasma and serum. All other patient groups represent different patients. All samples were diluted 4-fold with sample diluent prior to data acquisition.

The fluorescence responses and concentrations of analytes were obtained using a Bio-Plex Pro™ Human Inflammation Panel 37-Plex assay kit with magnetic beads (171AL1001M, Bio-Rad, Hercules, California, USA) and analysed with a Luminex100 system and the accompanying Bio-Plex Manager^TM^ Software 6.1(Bio-Rad, Hercules, California, USA).

The concentration values and detection limits were determined from standard curves generated from each kit’s standards using the Bio-Plex Software Manager^TM^ weighted 5PL curve fitting procedure. To maximize the number of concentrations values available for analysis we included the Bio-Plex extrapolated values. Therefore, the definition of out-of-range here, and unless otherwise stated, refers to concentration values that cannot be obtained from the 5PL logistic curve; that is beyond extrapolation.

All statistical analysis was performed using R version 3.1.0 (2014-04-10)^45^ via RStudio Version 0.98.507^46^. The mixed-effects modelling were done using lmer^47^. The visualization of regression results was done using visreg^44^, and the significance of interactions terms and interaction means were determined using Phia package^43^. Unless otherwise stated all *p-values* have been multiple test corrected according to Holm’s method^48^. For simulation experiments normal distributions were obtained from rnorm and skewed distribution were obtained using skew normal distribution methods, rsn, from the R package sn^49^.

## Additional Information

**How to cite this article**: Breen, E. J. *et al.* The Statistical Value of Raw Fluorescence Signal in Luminex xMAP Based Multiplex Immunoassays. *Sci. Rep.* **6**, 26996; doi: 10.1038/srep26996 (2016).

## Supplementary Material

Supplementary Information

## Figures and Tables

**Table 1:** Comparison of point scatter above and below the level of detection (LOD) for various analytes and tissues combinations.

Cytokine	Tissue	LOD	N	Above	Below	CV-A	CV-B	Ratio	p.value	Sig.
IFN-g (21)	plasma	17.418	11	80	11	0.025	0.076	0.327	0.001	**
IFN-g (21)	serum	17.418	28	52	28	0.059	0.081	0.724	0.064	
IL-10 (56)	plasma	34.966	29	29	62	0.161	0.129	1.248	0.472	
IL-10 (56)	serum	34.966	32	48	32	0.116	0.133	0.873	0.319	
IL-11 (39)	plasma	27.995	33	33	58	0.14	0.123	1.136	0.945	
IL-11 (39)	serum	27.995	37	37	43	1.239	0.146	8.469	0	***
IL-22 (18)	plasma	27.463	12	79	12	0.016	0.092	0.173	0	***
IL-22 (18)	serum	27.463	24	56	24	0.082	0.108	0.759	0.188	
IL-27 (13)	plasma	24.529	9	82	9	0.027	0.101	0.271	0.001	**
IL-27 (13)	serum	24.529	23	56	23	0.157	0.155	1.013	0.461	
IL-29 (33)	serum	18.187	19	61	19	0.045	0.078	0.574	0.018	*
IL-32 (35)	plasma	58.531	37	37	54	0.262	0.204	1.282	0.867	
IL-32 (35)	serum	58.531	39	41	39	0.322	0.284	1.131	0.902	
IL-34 (15)	plasma	60.664	16	16	75	0.229	0.177	1.296	0.868	
IL-34 (15)	serum	60.664	24	24	56	1.319	0.176	7.509	0	***
Light (51)	plasma	136.28	29	62	29	0.059	0.116	0.505	0.001	**
Light (51)	serum	136.28	8	72	8	0.049	0.106	0.465	0.056	

**LOD** represents the fluorescence level associated with the concentration determined as the lower level of detection, N the number of responses above and below the associated **LOD** used in the analysis. **Above** and **Below** give the actual number of observed responses above and below the **LOD**. The columns **CV-A** and **CV-B** give the coefficient of variation (CV) of the samples, each of size **N**, above and below the respective **LOD. Ratio** gives the value of **CV-A/CV-B. P.value** represents the uncorrected probability of an **F test** statistic. The number of *’s in the **Sig.** column indicates the significance of the associated *p-values* for each analyte and tissue combination.

**Table 2:** Comparison of analyte expression across tissue types.

Analyte	Fluorescence			Concentration		
	Chisq	Pr(>Chisq)	Sig.	Chisq	Pr(>Chisq)	Sig.
April (42)	29.9083	3.17E-05	***	16.7169	0.007832	**
Baff (37)	113.643	<2.2e-16	***	62.002	4.39E-12	***
CD163 (46)	238.8243	<2.2e-16	***	203.1032	<2.2e-16	***
CD30 (53)	211.2805	<2.2e-16	***	85.6809	<2.2e-16	***
Chitinase (72)	424.5781	<2.2e-16	***	317.3054	<2.2e-16	***
gp130 (14)	103.0032	<2.2e-16	***	131.1003	<2.2e-16	***
IFN-a2 (20)	6.3883	0.84757		16.7829	0.007832	**
IFN-b (44)	7.8082	0.5516		11.4468	0.047698	*
IFN-g (21)	8.8317	0.41099		33.9826	3.40E-06	***
IL-10 (56)	3.907	1		4.4864	0.640518	
IL-11 (39)	0.8161	1				
IL-12p40 (28)	3.2292	1		12.739	0.031419	*
IL-12p70 (75)	1.0107	1		1.356	1	
IL-19 (29)	358.3435	<2.2e-16	***	167.5504	<2.2e-16	***
IL-2 (38)	2.5195	1		35.0154	2.18E-06	***
IL-20 (30)	8.292	0.48416		17.9174	0.005032	**
IL-22 (18)	14.932	0.03001	*	29.4719	2.85E-05	***
IL-26 (22)	12.2377	0.09918	.	13.608	0.024432	*
IL-27 (13)	16.4034	0.01781	*	20.5327	0.00158	**
IL-28 (66)	1.7238	1		10.1141	0.070482	.
IL-29 (33)	5.8678	0.9458		26.1033	0.000127	***
IL-32 (35)	76.6646	3.66E-15	***	73.4943	1.60E-14	***
IL-34 (15)	11.9239	0.10708				
IL-35 (34)	15.7757	0.02143	*	15.6706	0.010597	*
IL-6RA (19)	233.956	<2.2e-16	***	208.3386	<2.2e-16	***
IL-8 (54)	544.3097	<2.2e-16	***	223.1665	<2.2e-16	***
Light (51)	29.2716	4.12E-05	***			
MMP-1 (43)	7.5231	0.56967		56.0105	7.94E-11	***
MMP-2 (26)	244.5459	<2.2e-16	***	180.0698	<2.2e-16	***
MMP-3 (45)	228.2042	<2.2e-16	***			
OCN (65)	255.3559	<2.2e-16	***	233.1102	<2.2e-16	***
OPN (77)	717.7917	<2.2e-16	***	225.871	<2.2e-16	***
Pentraxin (48)	131.4268	<2.2e-16	***	136.3573	<2.2e-16	***
TNFR1 (73)	28.7805	4.98E-05	***	29.273	2.94E-05	***
TNFR2 (67)	274.9578	<2.2e-16	***	193.6078	<2.2e-16	***
TSLP (52)	1.3719	1		0.5499	1	
Tweak (62)	16.2958	0.01781	*	21.084	0.001315	**

The probability values (Pr(>Chisq)) of the Chi-square result have been multiple test corrected using Holm’s methods^48^. The number of *’s in the Sig. column gives an indication of the analytes significance across each tissue. Note the empty rows associated with the concentrations results represents the analyte that couldn’t be analysed because of missing values. Degrees of freedom for all tests is 3.

**Table 3:** Significant analyte pair-wise condition (contrast) differences.

Analyte	Contrast	Fluorescence				Concentration			
		Value	Chisq	Pr(>Chisq)	Sig.	Value	Chisq	Pr(>Chisq)	Sig
April (42)	Normal-COPD	−0.908	14.060	0.040	*	−0.656	4.678	1.000	
April (42)	Normal-Mono	1.383	69.060	0.000	***	1.484	54.024	0.000	***
Baff (37)	Normal-Mono	−0.907	29.659	0.000	***	−0.319	2.492	1.000	
CD163 (46)	Normal-Mono	−0.775	21.684	0.001	***	−0.760	14.180	0.032	*
CD30 (53)	Normal-Mono	−0.878	27.833	0.000	***	−0.861	18.190	0.004	**
IL-20 (30)	Normal-Mono	−0.398	5.717	1.000		−0.779	14.901	0.022	*
IL-26 (22)	Normal-Mono	−0.695	17.416	0.007	**	−1.031	26.082	0.000	***
IL-27 (13)	Normal-Mono	−1.417	71.749	0.000	***	−1.796	58.714	0.000	***
MMP-1 (43)	Normal-Mono	0.341	4.204	1.000		1.550	49.783	0.000	***
MMP-3 (45)	Normal-Mono	1.043	39.227	0.000	***				
OCN (65)	Normal-Mono	0.645	15.010	0.024	*	0.468	5.368	1.000	
OPN (77)	Normal-Mono	0.948	32.415	0.000	***	0.841	17.349	0.006	**
TNFR2 (67)	Normal-Mono	−0.741	19.828	0.002	**	−0.606	9.008	0.473	
April (42)	Normal-Myeloma	1.831	70.681	0.000	***	2.055	53.370	0.000	***
IL-32 (35)	Normal-Myeloma	−1.014	21.689	0.001	***	−1.428	16.454	0.010	**
MMP-1 (43)	Normal-Myeloma	0.317	2.114	1.000		1.529	24.371	0.000	***
OCN (65)	Normal-Myeloma	0.804	13.632	0.049	*	0.760	7.302	1.000	
OPN (77)	Normal-Myeloma	3.864	314.651	0.000	***	2.665	89.768	0.000	***
MMP-1 (43)	Normal-Psoriasis	−1.286	29.022	0.000	***	−1.945	41.329	0.000	***
OPN (77)	Normal-Psoriasis	1.669	48.862	0.000	***	1.067	12.881	0.063	.
April (42)	Normal-RA	−0.737	13.852	0.044	*	−0.759	10.660	0.198	
IFN-a2 (20)	Normal-RA	−0.512	6.689	1.000		−1.025	19.217	0.002	**
IFN-b (44)	Normal-RA	−0.750	14.350	0.034	*	−1.334	32.520	0.000	***
IFN-g (21)	Normal-RA	−0.194	0.958	1.000		−1.256	13.492	0.046	*
IL-10 (56)	Normal-RA	−1.357	47.009	0.000	***	−1.789	31.465	0.000	***
IL-11 (39)	Normal-RA	−1.140	33.178	0.000	***				
IL-12p40 (28)	Normal-RA	−0.608	9.423	0.450		−1.413	36.518	0.000	***
IL-12p70 (75)	Normal-RA	−1.481	56.015	0.000	***	−2.429	107.861	0.000	***
IL-19 (29)	Normal-RA	−1.152	33.862	0.000	***	−1.218	27.458	0.000	***
IL-2 (38)	Normal-RA	−1.037	27.469	0.000	***	−2.470	105.001	0.000	***
IL-20 (30)	Normal-RA	−1.563	62.396	0.000	***	−2.669	131.993	0.000	***
IL-22 (18)	Normal-RA	−1.722	75.713	0.000	***	−3.814	148.767	0.000	***
IL-26 (22)	Normal-RA	−0.536	7.335	1.000		−0.959	17.045	0.007	**
IL-27 (13)	Normal-RA	−1.532	59.846	0.000	***	−2.300	65.341	0.000	***
IL-28 (66)	Normal-RA	−0.543	7.523	1.000		−1.271	29.110	0.000	***
IL-29 (33)	Normal-RA	−0.621	9.838	0.362		−2.215	53.627	0.000	***
IL-35 (34)	Normal-RA	−1.006	25.855	0.000	***	−1.432	37.969	0.000	***
IL-8 (54)	Normal-RA	−1.320	44.442	0.000	***	−1.076	21.460	0.001	***
TSLP (52)	Normal-RA	−0.633	10.234	0.295		−1.225	27.796	0.000	***
April (42)	Normal -Sepsis	−1.106	22.188	0.001	***	−0.747	6.568	1.000	
Baff (37)	Normal -Sepsis	−1.795	58.436	0.000	***	−1.115	14.626	0.025	*
Chitinase (72)	Normal -Sepsis	−0.530	5.087	1.000		−1.111	13.591	0.043	*
IL-10 (56)	Normal -Sepsis	−0.870	13.724	0.047	*	−0.903	6.364	1.000	
IL-2 (38)	Normal -Sepsis	−0.311	1.753	1.000		−1.314	19.372	0.002	**
IL-8 (54)	Normal -Sepsis	−2.288	94.906	0.000	***	−1.581	29.423	0.000	***
MMP-1 (43)	Normal -Sepsis	−2.248	91.631	0.000	***	−2.083	48.468	0.000	***
MMP-3 (45)	Normal -Sepsis	−0.903	14.798	0.027	*				
Pentraxin (48)	Normal -Sepsis	−0.952	16.419	0.012	*	−0.860	8.694	0.555	
TNFR1 (73)	Normal -Sepsis	−0.975	17.250	0.008	**	−1.355	21.622	0.001	***
TNFR2 (67)	Normal -Sepsis	−1.282	29.817	0.000	***	−1.611	30.543	0.000	***
April (42)	Normal -T2D	−0.751	17.352	0.007	**	−0.600	8.819	0.522	
IFN-g (21)	Normal -T2D	−0.329	3.340	1.000		−1.090	23.031	0.000	***
IL-12p40 (28)	Normal -T2D	−0.184	1.044	1.000		−0.827	16.684	0.009	**
IL-2 (38)	Normal -T2D	−0.094	0.272	1.000		−0.756	13.738	0.040	*
IL-28 (66)	Normal -T2D	−0.222	1.510	1.000		−0.807	15.846	0.013	*
IL-29 (33)	Normal -T2D	−0.256	2.022	1.000		−1.044	21.131	0.001	***

The probability values (Pr(>Chisq)) of the Chi-square result have been multiple test corrected using Holm’s methods^48^. The number of *’s in the Sig. column indicates significance levels. Note the empty rows associated with the concentrations results represents analyte comparisons that couldn’t be analysed because of missing concentration values. COPD = chronic obstructive pulmonary disease, Mono = mononucleosis, RA = rheumatoid arthritis, T2D = type 2 diabetes. Degrees of freedom = 1 for all tests.
