# Supplementary material for: The Statistical Value of Raw Fluorescence Signal in Luminex xMAP Based Multiplex Immunoassays
Source: Sci Rep. 2016 May 31;6:26996. doi: 10.1038/srep26996 (PMC4886638; doi:10.1038/srep26996)
Supplement: Supplementary Information [file srep26996-s1.pdf]

Supplementary Information for

The Statistical Value of Raw Fluorescence Signal in Luminex xMAP Based Multiplex Immunoassays.

By Edmond J. Breen[1], Woei Tan[2], and Alamgir Khan[1]

[1]Australian Proteome Analysis Facility (APAF), Level 4, Building F7B, Research Park Drive, Macquarie University, Sydney NSW 2109 Australia.

[2]Bio-Rad Laboratories Inc. 2000 Alfred Nobel Drive, Hercules, CA 94547. USA

Table S1: Patient numbers according to plate, tissue and condition groupings. COPD= chronic obstructive pulmonary disease, Mono = mononucleosis, RA = rheumatoid arthritis, T2D = type 2 diabetes. Note, the 16 mononucleosis patients across plasma and serum are paired, as is the 6 patients with respect to myeloma. All other patient groups represent different people.

			Condition								
		**Plate**	**COPD**	**Mono**	**Myeloma**	**Normal**	**Psoriasis**	**RA**	**Sepsis**	**T2D**	Total
**Tissue**	**plasma**	**1**				16	10			43	69
	**saliva**										
	**serum**		9								9
	**urine**										
	**plasma**	**2**									
	**saliva**										
	**serum**					17		20			37
	**urine**										
	**plasma**	3		16	6						22
	**saliva**					10					10
	**serum**			16	6	2			10		34
	**urine**					10					10
	Total		9	32	12	55	10	20	10	43	191

Table S2: Fixed analyte, pairwise tissue comparisons.

			Fluorescence				Concentration			
**Analyte**	**Contrast**	**Df**	**Value**	**Chisq**	**Pr(>Chisq)**	**Sig.**	**Value**	**Chisq**	**Pr(>Chisq)**	**Sig.**
April (42)	plasma-saliva	1	0.817	5.860	1.000		1.026	5.946	1.000	
Baff (37)	plasma-saliva	1	1.069	10.031	0.223		1.218	8.394	0.399	
CD163 (46)	plasma-saliva	1	4.392	169.203	0.000	***	5.189	142.799	0.000	***
CD30 (53)	plasma-saliva	1	3.865	131.001	0.000	***	5.990	60.672	0.000	***
Chitinase (72)	plasma-saliva	1	-0.512	2.296	1.000		-0.310	0.542	1.000	
gp130 (14)	plasma-saliva	1	-0.031	0.008	1.000		0.102	0.059	1.000	
IFN-a2 (20)	plasma-saliva	1	-0.543	2.590	1.000		-0.722	2.945	1.000	
IFN-b (44)	plasma-saliva	1	-0.563	2.776	1.000		-0.447	1.129	1.000	
IFN-g (21)	plasma-saliva	1	-0.191	0.318	1.000		0.720	1.945	1.000	
IL-10 (56)	plasma-saliva	1	-0.600	3.156	1.000		-0.421	0.762	1.000	
IL-11 (39)	plasma-saliva	1	-0.214	0.401	1.000					
IL-12p40 (28)	plasma-saliva	1	-0.430	1.623	1.000		-0.361	0.692	1.000	
IL-12p70 (75)	plasma-saliva	1	-0.235	0.486	1.000		-0.322	0.550	1.000	
IL-19 (29)	plasma-saliva	1	-6.041	320.048	0.000	***	-5.332	160.713	0.000	***
IL-2 (38)	plasma-saliva	1	-0.066	0.038	1.000		0.419	0.860	1.000	
IL-20 (30)	plasma-saliva	1	-0.595	3.104	1.000		-0.756	3.229	1.000	
IL-22 (18)	plasma-saliva	1	-0.853	6.378	1.000		-1.595	12.971	0.040	*
IL-26 (22)	plasma-saliva	1	-1.079	10.221	0.204		-1.371	10.630	0.128	
IL-27 (13)	plasma-saliva	1	-0.320	0.895	1.000		0.423	0.910	1.000	
IL-28 (66)	plasma-saliva	1	-0.067	0.039	1.000		-0.046	0.011	1.000	
IL-29 (33)	plasma-saliva	1	-0.321	0.904	1.000		-1.393	8.069	0.459	
IL-32 (35)	plasma-saliva	1	-1.269	14.118	0.026	*	-2.860	24.394	0.000	***
IL-34 (15)	plasma-saliva	1	-0.878	6.769	1.000					
IL-35 (34)	plasma-saliva	1	-1.284	14.452	0.022	*	-1.473	12.265	0.056	.
IL-6RA (19)	plasma-saliva	1	4.286	161.115	0.000	***	4.999	141.295	0.000	***
IL-8 (54)	plasma-saliva	1	-7.391	479.089	0.000	***	-5.954	200.424	0.000	***
Light (51)	plasma-saliva	1	0.107	0.101	1.000					
MMP-1 (43)	plasma-saliva	1	-0.323	0.917	1.000		-0.517	1.374	1.000	
MMP-2 (26)	plasma-saliva	1	2.890	73.235	0.000	***	3.552	62.163	0.000	***
MMP-3 (45)	plasma-saliva	1	3.480	106.228	0.000	***				
OCN (65)	plasma-saliva	1	4.475	175.641	0.000	***	7.731	179.736	0.000	***
OPN (77)	plasma-saliva	1	8.552	641.545	0.000	***	6.195	203.495	0.000	***
Pentraxin (48)	plasma-saliva	1	-1.421	17.708	0.004	**	-1.260	8.972	0.302	
TNFR1 (73)	plasma-saliva	1	0.017	0.003	1.000		0.112	0.071	1.000	
TNFR2 (67)	plasma-saliva	1	4.104	147.705	0.000	***	4.623	113.354	0.000	***
TSLP (52)	plasma-saliva	1	-0.235	0.485	1.000		0.261	0.361	1.000	
Tweak (62)	plasma-saliva	1	-0.176	0.271	1.000		0.301	0.513	1.000	
April (42)	plasma-serum	1	1.027	28.363	0.000	***	0.808	12.389	0.053	.
Baff (37)	plasma-serum	1	-0.583	9.151	0.351		-0.285	1.544	1.000	
CD163 (46)	plasma-serum	1	-0.367	3.613	1.000		-0.310	1.822	1.000	
CD30 (53)	plasma-serum	1	-0.277	2.057	1.000		-0.308	1.806	1.000	
Chitinase (72)	plasma-serum	1	-0.614	10.124	0.214		-0.630	7.432	0.634	
gp130 (14)	plasma-serum	1	-0.397	4.250	1.000		-0.582	6.426	1.000	
IFN-a2 (20)	plasma-serum	1	0.116	0.361	1.000		0.498	4.671	1.000	
IFN-b (44)	plasma-serum	1	-0.050	0.068	1.000		-0.181	0.614	1.000	
IFN-g (21)	plasma-serum	1	0.234	1.470	1.000		1.781	22.662	0.000	***
IL-10 (56)	plasma-serum	1	-0.044	0.052	1.000		-0.411	1.800	1.000	
IL-11 (39)	plasma-serum	1	0.075	0.152	1.000					
IL-12p40 (28)	plasma-serum	1	0.149	0.596	1.000		0.705	9.357	0.249	
IL-12p70 (75)	plasma-serum	1	0.067	0.119	1.000		0.089	0.150	1.000	
IL-19 (29)	plasma-serum	1	-0.087	0.203	1.000		-0.369	2.591	1.000	
IL-2 (38)	plasma-serum	1	0.150	0.605	1.000		1.002	18.098	0.003	**
IL-20 (30)	plasma-serum	1	0.018	0.009	1.000		0.232	1.022	1.000	
IL-22 (18)	plasma-serum	1	0.145	0.562	1.000		0.284	0.977	1.000	
IL-26 (22)	plasma-serum	1	-0.119	0.380	1.000		-0.115	0.252	1.000	
IL-27 (13)	plasma-serum	1	0.493	6.471	1.000		0.956	12.880	0.041	*
IL-28 (66)	plasma-serum	1	0.203	1.110	1.000		0.617	7.097	0.741	

IL-29 (33)	plasma-serum	1	0.228	1.402	1.000		0.616	5.147	1.000	
IL-32 (35)	plasma-serum	1	0.152	0.619	1.000		-0.160	0.259	1.000	
IL-34 (15)	plasma-serum	1	-0.140	0.525	1.000					
IL-35 (34)	plasma-serum	1	-0.021	0.012	1.000		0.148	0.417	1.000	
IL-6RA (19)	plasma-serum	1	-0.447	5.371	1.000		-0.506	4.864	1.000	
IL-8 (54)	plasma-serum	1	-0.301	2.441	1.000		-0.445	3.761	1.000	
Light (51)	plasma-serum	1	-0.128	0.440	1.000					
MMP-1 (43)	plasma-serum	1	-0.473	6.022	1.000		-1.677	42.490	0.000	***
MMP-2 (26)	plasma-serum	1	-1.470	58.131	0.000	***	-1.220	28.236	0.000	***
MMP-3 (45)	plasma-serum	1	-0.528	7.491	0.843					
OCN (65)	plasma-serum	1	0.008	0.002	1.000		0.076	0.110	1.000	
OPN (77)	plasma-serum	1	1.234	40.934	0.000	***	0.854	13.849	0.026	*
Pentraxin (48)	plasma-serum	1	0.258	1.789	1.000		0.324	1.990	1.000	
TNFR1 (73)	plasma-serum	1	-0.633	10.790	0.151		-0.741	10.430	0.141	
TNFR2 (67)	plasma-serum	1	-0.833	18.673	0.002	**	-0.758	10.902	0.112	
TSLP (52)	plasma-serum	1	0.005	0.001	1.000		0.025	0.012	1.000	
Tweak (62)	plasma-serum	1	-0.234	1.469	1.000		-0.128	0.310	1.000	
April (42)	plasma-urine	1	0.924	7.491	0.843		1.173	7.781	0.528	
Baff (37)	plasma-urine	1	2.892	73.339	0.000	***	2.859	46.198	0.000	***
CD163 (46)	plasma-urine	1	2.590	58.822	0.000	***	3.176	57.024	0.000	***
CD30 (53)	plasma-urine	1	-1.450	18.447	0.003	**	-1.477	12.333	0.054	.
Chitinase (72)	plasma-urine	1	5.959	311.470	0.000	***	6.529	240.668	0.000	***
gp130 (14)	plasma-urine	1	2.915	74.548	0.000	***	4.105	95.260	0.000	***
IFN-a2 (20)	plasma-urine	1	-0.567	2.818	1.000		-0.895	4.519	1.000	
IFN-b (44)	plasma-urine	1	-0.848	6.303	1.000		-1.419	11.362	0.090	.
IFN-g (21)	plasma-urine	1	-0.733	4.715	1.000		-0.897	4.079	1.000	
IL-10 (56)	plasma-urine	1	-0.408	1.462	1.000		0.445	0.800	1.000	
IL-11 (39)	plasma-urine	1	-0.073	0.047	1.000					
IL-12p40 (28)	plasma-urine	1	-0.129	0.145	1.000		0.112	0.070	1.000	
IL-12p70 (75)	plasma-urine	1	-0.157	0.215	1.000		-0.276	0.430	1.000	
IL-19 (29)	plasma-urine	1	-0.552	2.675	1.000		-0.931	4.902	1.000	
IL-2 (38)	plasma-urine	1	-0.359	1.129	1.000		-1.140	7.285	0.682	
IL-20 (30)	plasma-urine	1	-0.810	5.760	1.000		-1.412	11.275	0.093	.
IL-22 (18)	plasma-urine	1	-0.899	7.086	1.000		-2.091	22.300	0.000	***
IL-26 (22)	plasma-urine	1	-0.729	4.666	1.000		-0.989	5.529	1.000	
IL-27 (13)	plasma-urine	1	-0.665	3.879	1.000		-0.749	2.857	1.000	
IL-28 (66)	plasma-urine	1	-0.102	0.091	1.000		-0.325	0.596	1.000	
IL-29 (33)	plasma-urine	1	-0.475	1.981	1.000		-1.599	13.059	0.038	*
IL-32 (35)	plasma-urine	1	-2.614	59.937	0.000	***	-4.303	63.683	0.000	***
IL-34 (15)	plasma-urine	1	-0.973	8.310	0.540					
IL-35 (34)	plasma-urine	1	-0.356	1.109	1.000		-0.123	0.086	1.000	
IL-6RA (19)	plasma-urine	1	2.499	54.759	0.000	***	2.955	49.372	0.000	***
IL-8 (54)	plasma-urine	1	-0.065	0.037	1.000		0.089	0.044	1.000	
Light (51)	plasma-urine	1	-1.691	25.095	0.000	***				
MMP-1 (43)	plasma-urine	1	0.101	0.090	1.000		0.601	1.972	1.000	
MMP-2 (26)	plasma-urine	1	2.343	48.140	0.000	***	3.116	51.532	0.000	***
MMP-3 (45)	plasma-urine	1	3.430	103.216	0.000	***				
OCN (65)	plasma-urine	1	3.495	107.127	0.000	***	3.559	71.622	0.000	***
OPN (77)	plasma-urine	1	-0.126	0.138	1.000		-0.974	2.858	1.000	
Pentraxin (48)	plasma-urine	1	2.976	77.669	0.000	***	4.174	98.502	0.000	***
TNFR1 (73)	plasma-urine	1	-1.534	20.642	0.001	***	-1.919	20.811	0.001	***
TNFR2 (67)	plasma-urine	1	-1.660	24.179	0.000	***	-1.695	16.243	0.008	**
TSLP (52)	plasma-urine	1	-0.336	0.989	1.000		-0.104	0.061	1.000	
Tweak (62)	plasma-urine	1	1.059	9.828	0.246		1.751	17.330	0.004	**
April (42)	saliva-serum	1	0.210	0.398	1.000		-0.218	0.279	1.000	
Baff (37)	saliva-serum	1	-1.653	24.765	0.000	***	-1.504	13.303	0.034	*
CD163 (46)	saliva-serum	1	-4.759	205.310	0.000	***	-5.499	166.467	0.000	***
CD30 (53)	saliva-serum	1	-4.141	155.488	0.000	***	-6.299	67.875	0.000	***
Chitinase (72)	saliva-serum	1	-0.102	0.094	1.000		-0.320	0.603	1.000	
gp130 (14)	saliva-serum	1	-0.367	1.219	1.000		-0.684	2.754	1.000	
IFN-a2 (20)	saliva-serum	1	0.659	3.940	1.000		1.221	8.693	0.342	
IFN-b (44)	saliva-serum	1	0.512	2.380	1.000		0.266	0.414	1.000	
IFN-g (21)	saliva-serum	1	0.424	1.632	1.000		1.061	3.284	1.000	

IL-10 (56)	saliva-serum	1	0.556	2.799	1.000		0.010	0.000	1.000	
IL-11 (39)	saliva-serum	1	0.289	0.758	1.000					
IL-12p40 (28)	saliva-serum	1	0.579	3.040	1.000		1.067	6.215	1.000	
IL-12p70 (75)	saliva-serum	1	0.302	0.827	1.000		0.412	0.925	1.000	
IL-19 (29)	saliva-serum	1	5.954	321.390	0.000	***	4.962	144.885	0.000	***
IL-2 (38)	saliva-serum	1	0.216	0.422	1.000		0.582	1.660	1.000	
IL-20 (30)	saliva-serum	1	0.613	3.406	1.000		0.988	5.741	1.000	
IL-22 (18)	saliva-serum	1	0.997	9.018	0.374		1.879	14.220	0.021	*
IL-26 (22)	saliva-serum	1	0.961	8.368	0.527		1.256	9.281	0.257	
IL-27 (13)	saliva-serum	1	0.812	5.981	1.000		0.533	1.326	1.000	
IL-28 (66)	saliva-serum	1	0.270	0.662	1.000		0.663	2.380	1.000	
IL-29 (33)	saliva-serum	1	0.549	2.735	1.000		2.009	14.819	0.016	*
IL-32 (35)	saliva-serum	1	1.420	18.294	0.003	**	2.700	24.064	0.000	***
IL-34 (15)	saliva-serum	1	0.739	4.948	1.000					
IL-35 (34)	saliva-serum	1	1.263	14.452	0.022	*	1.621	15.462	0.011	*
IL-6RA (19)	saliva-serum	1	-4.733	203.083	0.000	***	-5.505	178.334	0.000	***
IL-8 (54)	saliva-serum	1	7.089	455.675	0.000	***	5.509	178.569	0.000	***
Light (51)	saliva-serum	1	-0.235	0.502	1.000					
MMP-1 (43)	saliva-serum	1	-0.150	0.203	1.000		-1.160	7.053	0.752	
MMP-2 (26)	saliva-serum	1	-4.360	172.328	0.000	***	-4.772	116.128	0.000	***
MMP-3 (45)	saliva-serum	1	-4.008	145.636	0.000	***				
OCN (65)	saliva-serum	1	-4.467	180.877	0.000	***	-7.655	179.959	0.000	***
OPN (77)	saliva-serum	1	-7.319	485.638	0.000	***	-5.341	157.013	0.000	***
Pentraxin (48)	saliva-serum	1	1.679	25.552	0.000	***	1.583	14.752	0.016	*
TNFR1 (73)	saliva-serum	1	-0.650	3.835	1.000		-0.853	4.284	1.000	
TNFR2 (67)	saliva-serum	1	-4.937	220.978	0.000	***	-5.381	159.409	0.000	***
TSLP (52)	saliva-serum	1	0.240	0.522	1.000		-0.236	0.306	1.000	
Tweak (62)	saliva-serum	1	-0.058	0.031	1.000		-0.429	1.084	1.000	
April (42)	saliva-urine	1	0.107	0.073	1.000		0.148	0.087	1.000	
Baff (37)	saliva-urine	1	1.822	21.176	0.001	***	1.640	10.722	0.123	
CD163 (46)	saliva-urine	1	-1.802	20.721	0.001	***	-2.013	15.436	0.011	*
CD30 (53)	saliva-urine	1	-5.315	180.156	0.000	***	-7.467	83.794	0.000	***
Chitinase (72)	saliva-urine	1	6.471	267.040	0.000	***	6.839	186.403	0.000	***
gp130 (14)	saliva-urine	1	2.946	55.358	0.000	***	4.002	63.852	0.000	***
IFN-a2 (20)	saliva-urine	1	-0.023	0.003	1.000		-0.172	0.118	1.000	
IFN-b (44)	saliva-urine	1	-0.285	0.519	1.000		-0.972	3.762	1.000	
IFN-g (21)	saliva-urine	1	-0.543	1.878	1.000		-1.617	8.163	0.445	
IL-10 (56)	saliva-urine	1	0.192	0.234	1.000		0.866	2.597	1.000	
IL-11 (39)	saliva-urine	1	0.141	0.126	1.000					
IL-12p40 (28)	saliva-urine	1	0.302	0.580	1.000		0.473	0.852	1.000	
IL-12p70 (75)	saliva-urine	1	0.079	0.040	1.000		0.046	0.008	1.000	
IL-19 (29)	saliva-urine	1	5.488	192.109	0.000	***	4.400	77.182	0.000	***
IL-2 (38)	saliva-urine	1	-0.293	0.548	1.000		-1.559	8.775	0.330	
IL-20 (30)	saliva-urine	1	-0.215	0.296	1.000		-0.656	1.718	1.000	
IL-22 (18)	saliva-urine	1	-0.046	0.014	1.000		-0.496	0.982	1.000	
IL-26 (22)	saliva-urine	1	0.350	0.782	1.000		0.382	0.583	1.000	
IL-27 (13)	saliva-urine	1	-0.346	0.762	1.000		-1.172	5.477	1.000	
IL-28 (66)	saliva-urine	1	-0.035	0.008	1.000		-0.279	0.297	1.000	
IL-29 (33)	saliva-urine	1	-0.154	0.152	1.000		-0.206	0.144	1.000	
IL-32 (35)	saliva-urine	1	-1.345	11.544	0.101		-1.443	7.053	0.752	
IL-34 (15)	saliva-urine	1	-0.095	0.057	1.000					
IL-35 (34)	saliva-urine	1	0.928	5.492	1.000		1.350	7.262	0.683	
IL-6RA (19)	saliva-urine	1	-1.787	20.373	0.001	**	-2.044	16.653	0.006	**
IL-8 (54)	saliva-urine	1	7.326	342.250	0.000	***	6.043	145.537	0.000	***
Light (51)	saliva-urine	1	-1.799	20.639	0.001	***				
MMP-1 (43)	saliva-urine	1	0.425	1.151	1.000		1.118	4.762	1.000	
MMP-2 (26)	saliva-urine	1	-0.547	1.907	1.000		-0.437	0.661	1.000	
MMP-3 (45)	saliva-urine	1	-0.050	0.016	1.000					
OCN (65)	saliva-urine	1	-0.980	6.127	1.000		-4.171	42.805	0.000	***
OPN (77)	saliva-urine	1	-8.678	480.277	0.000	***	-7.169	122.992	0.000	***
Pentraxin (48)	saliva-urine	1	4.397	123.282	0.000	***	5.434	117.685	0.000	***
TNFR1 (73)	saliva-urine	1	-1.551	15.344	0.014	*	-2.031	16.435	0.007	**
TNFR2 (67)	saliva-urine	1	-5.764	211.887	0.000	***	-6.318	152.033	0.000	***

TSLP (52)	saliva-urine	1	-0.101	0.065	1.000		-0.365	0.506	1.000	
Tweak (62)	saliva-urine	1	1.234	9.716	0.259		1.449	8.374	0.400	
April (42)	serum-urine	1	-0.103	0.096	1.000		0.365	0.786	1.000	
Baff (37)	serum-urine	1	3.475	109.479	0.000	***	3.144	58.151	0.000	***
CD163 (46)	serum-urine	1	2.956	79.232	0.000	***	3.486	71.488	0.000	***
CD30 (53)	serum-urine	1	-1.174	12.489	0.062	.	-1.169	8.034	0.464	
Chitinase (72)	serum-urine	1	6.573	391.671	0.000	***	7.159	301.307	0.000	***
gp130 (14)	serum-urine	1	3.313	99.505	0.000	***	4.687	129.234	0.000	***
IFN-a2 (20)	serum-urine	1	-0.683	4.225	1.000		-1.393	11.322	0.091	.
IFN-b (44)	serum-urine	1	-0.798	5.767	1.000		-1.238	8.944	0.304	
IFN-g (21)	serum-urine	1	-0.967	8.477	0.500		-2.678	26.200	0.000	***
IL-10 (56)	serum-urine	1	-0.364	1.202	1.000		0.855	2.592	1.000	
IL-11 (39)	serum-urine	1	-0.148	0.200	1.000					
IL-12p40 (28)	serum-urine	1	-0.278	0.698	1.000		-0.594	2.056	1.000	
IL-12p70 (75)	serum-urine	1	-0.223	0.452	1.000		-0.365	0.779	1.000	
IL-19 (29)	serum-urine	1	-0.465	1.964	1.000		-0.562	1.857	1.000	
IL-2 (38)	serum-urine	1	-0.509	2.347	1.000		-2.141	25.739	0.000	***
IL-20 (30)	serum-urine	1	-0.828	6.222	1.000		-1.644	15.907	0.009	**
IL-22 (18)	serum-urine	1	-1.043	9.871	0.242		-2.376	22.723	0.000	***
IL-26 (22)	serum-urine	1	-0.611	3.380	1.000		-0.874	4.490	1.000	
IL-27 (13)	serum-urine	1	-1.158	12.153	0.074	.	-1.706	13.552	0.030	*
IL-28 (66)	serum-urine	1	-0.305	0.845	1.000		-0.942	5.134	1.000	
IL-29 (33)	serum-urine	1	-0.704	4.488	1.000		-2.215	21.548	0.000	***
IL-32 (35)	serum-urine	1	-2.766	69.359	0.000	***	-4.144	66.420	0.000	***
IL-34 (15)	serum-urine	1	-0.834	6.301	1.000					
IL-35 (34)	serum-urine	1	-0.335	1.015	1.000		-0.271	0.433	1.000	
IL-6RA (19)	serum-urine	1	2.946	78.661	0.000	***	3.461	70.492	0.000	***
IL-8 (54)	serum-urine	1	0.236	0.506	1.000		0.534	1.676	1.000	
Light (51)	serum-urine	1	-1.564	22.164	0.000	***				
MMP-1 (43)	serum-urine	1	0.575	2.994	1.000		2.278	28.988	0.000	***
MMP-2 (26)	serum-urine	1	3.813	131.811	0.000	***	4.335	103.574	0.000	***
MMP-3 (45)	serum-urine	1	3.958	142.047	0.000	***				
OCN (65)	serum-urine	1	3.486	110.205	0.000	***	3.483	71.384	0.000	***
OPN (77)	serum-urine	1	-1.359	16.750	0.007	**	-1.828	10.279	0.152	
Pentraxin (48)	serum-urine	1	2.718	66.973	0.000	***	3.850	87.230	0.000	***
TNFR1 (73)	serum-urine	1	-0.901	7.355	0.896		-1.177	8.156	0.445	
TNFR2 (67)	serum-urine	1	-0.827	6.202	1.000		-0.937	5.168	1.000	
TSLP (52)	serum-urine	1	-0.341	1.052	1.000		-0.129	0.098	1.000	
Tweak (62)	serum-urine	1	1.292	15.141	0.015	*	1.879	20.766	0.001	***

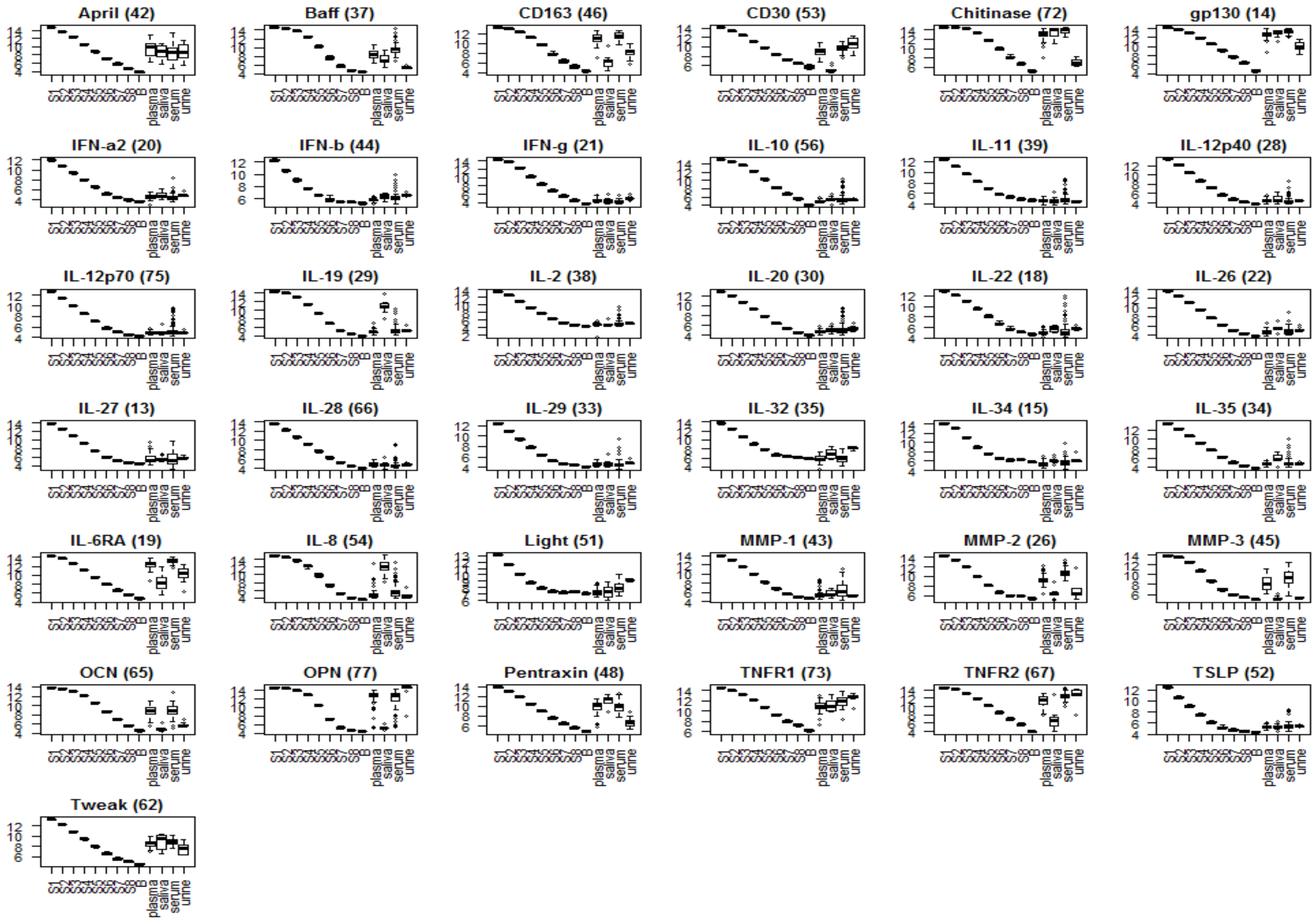

Figure S1: Distributions per analyte for test sample tissue types (plasma, saliva, serum and urine) together with their associated standards (S1, …, S8) and blank (B) readings. Y-axis represents the log2(FI).

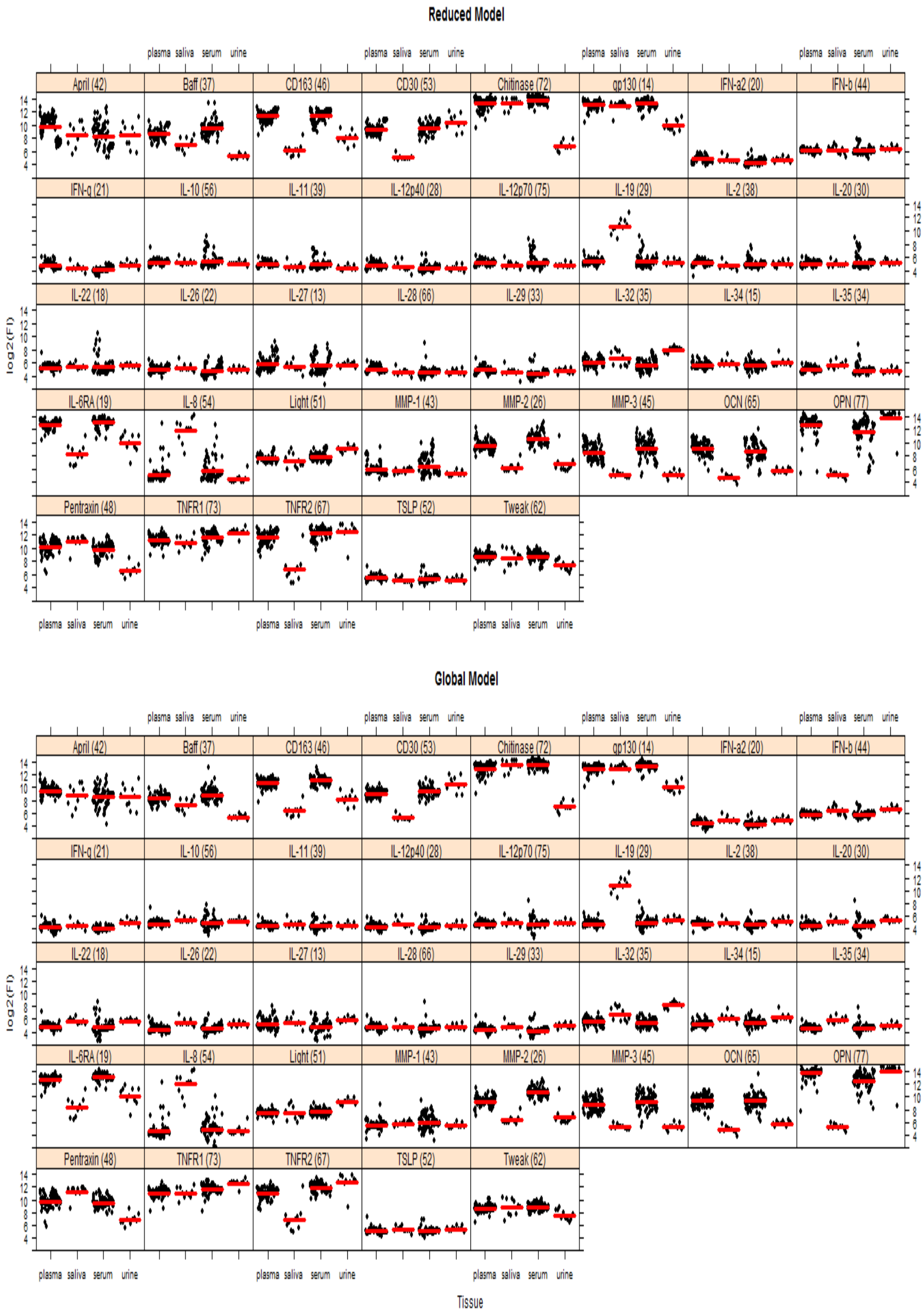

Figure S2: Comparison between global and reduced mixed-effects models. Note for the global model, the cluster of points (residuals) around each conditional mean response (red line) is tighter than that seen in the reduced model. Note both models contain the same number of samples per tissue (see main text for details).

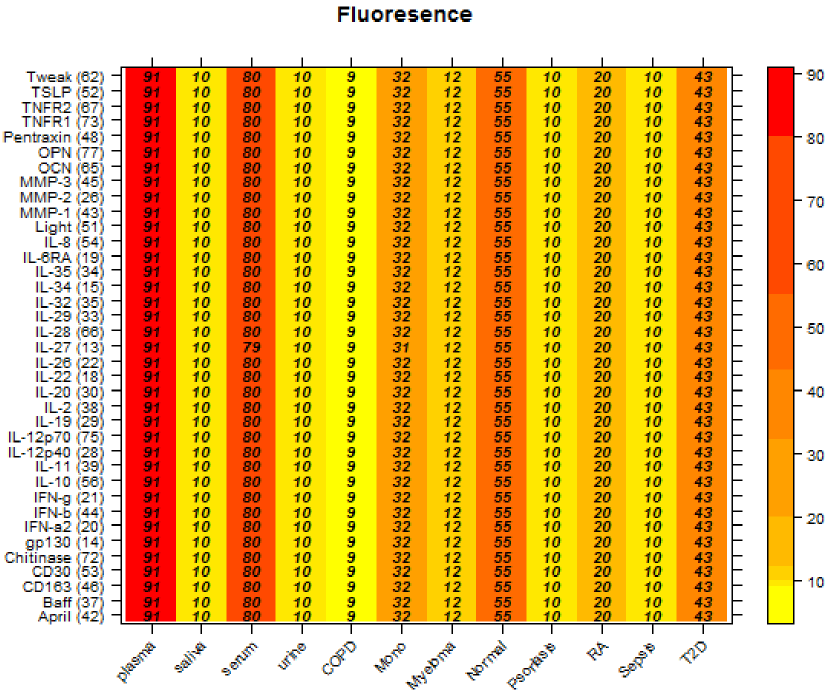

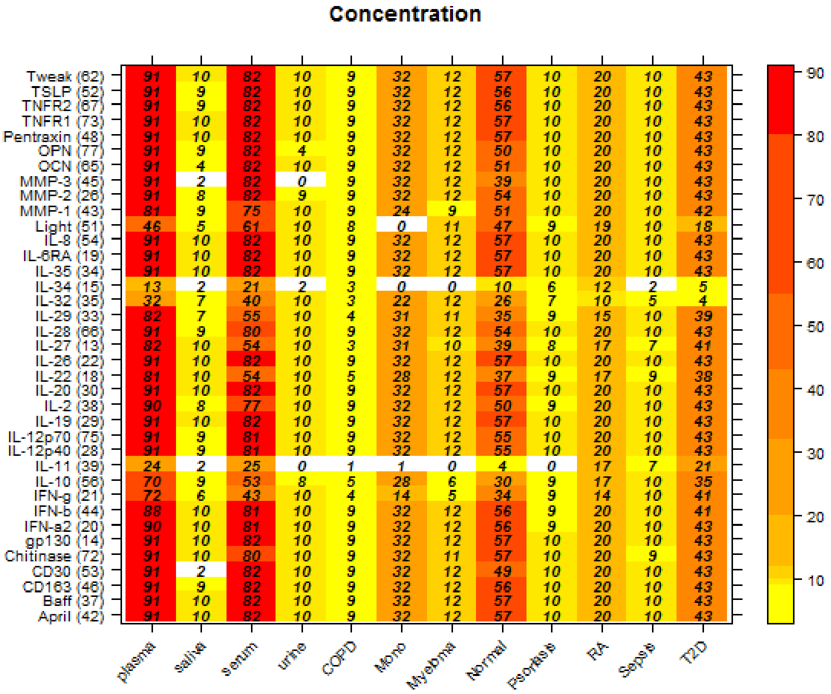

Figure S3: Arrays showing the number of fluorescence and concentration patient readings per analyte with respect to tissue and condition according to fluorescence or concentration data. Note for the fluorescence data there is one less reading (79) than expected (80) with respect to serum and IL-27. This is because one test for IL-27 in serum, failed to produce a fluorescence response. The fluorescence responses (FI) represent the median fluorescence (MFI) obtained from a set of individual beads, of the same type. The beads are detected and counted at the flow cytometer level and, per analyte per test and for the Bio-Plex Manager software; the absolute minimum number of beads to count per analyte for statistical meaningful values is 25 however, in practice all our assays are set up to count at least 50 beads. Abbreviations: COPD= chronic obstructive pulmonary disease, Mono = mononucleosis, RA = rheumatoid arthritis, T2D = type 2 diabetes.
